# Boundary violations and adolescent drinking: Observational evidence that symbolic boundaries moderate social influence

**DOI:** 10.1371/journal.pone.0224185

**Published:** 2019-11-05

**Authors:** Achim Edelmann

**Affiliations:** 1 Institute of Sociology, University of Bern, Bern, Switzerland; 2 Department of Sociology, London School of Economics and Political Science, London, England, United Kingdom; 3 Duke Network Analysis Center, Duke University, Durham, North Carolina, United States of America; Middlesex University, UNITED KINGDOM

## Abstract

Scholars of social influence can benefit from attending to symbolic boundaries. A common and influential way to understand symbolic boundaries is as widely shared understandings of what types of behaviors, tastes, and opinions are appropriate for different kinds of people. Scholars following this understanding have mostly focused on how people judge *others* and how symbolic boundaries align with and thus *reproduce* social differences. Although this work has been impressive, I argue that it might miss important ways in which symbolic boundaries become effective in everyday social life. I therefore develop an understanding of how symbolic boundaries affect people’s ideas and decisions about *themselves* and their *own* behavior. Based on this, I argue that focusing on *boundary violations*—that is, what happens if people express opinions or enact behavior that contravenes what is considered (in)appropriate for people like them—might offer an important way to understand how symbolic boundaries initiate and shape cultural and social change. Using data from Add Health, I demonstrate the utility of this line of argument and show that boundary violations play an important role in channeling social influence. Conservative/Evangelical Protestants and to a lesser degree Catholics, but not Mainline Protestants are highly influenced by the drinking of co-religionists. I consider the implications for cultural sociology.

## Introduction

Social influence is widespread. People adopt behavior and change opinions as a result of interacting with others (e.g. [1–3]). Studies have shown that such influence is stronger between people that share some characteristics (e.g., [4]) and scholars have argued that this is especially the case if people identify as being part of the same social group (e.g., [5–7], also [8,9]). However, this leaves us with no answers to the questions of which social identifications matter for the spread of what kind of behavior and how do they matter?

To gain leverage over these questions, I turn towards a concept from cultural sociology: symbolic boundaries. Sociologists have long argued that culture might moderate social influence [10,11]; also [12,13], yet those arguments have remained at a general level and explications of related mechanisms have remained vague. Symbolic boundaries enable theorizing such a mechanism because they account for the relation between group identifications and appropriate behavior, opinions, and tastes. This requires addressing two shortcomings in how scholars currently understand and use this concept.

One common and influential way to understand symbolic boundaries is as widely shared conceptual distinctions between kinds of people, practices and things (see [14]). These distinctions are based on subjective and intersubjective classifications [15] that are created, invoked, and negotiated in norms, cultural practices, and attitudes [16]. Classic examples include the distinction between the “sacred” and the “profane” [17], between the “pure” and the “impure” [18], or between different “status groups” [19].

This understanding of symbolic boundaries has proven useful to gain insights into a range of important issues. It has helped advance an understanding of how people create, maintain, and contest institutionalized social differences. To do so, scholars have focused on how people do “boundary work,” that is, how they produce, rationalize, and contest similarities and differences between social groups based on class, race, religion or gender (e.g., [20–24]). For example, Michèle Lamont has shown how symbolic boundaries can help to understand the status differences people draw between members of different social classes [25]. Others have used the concept to understand how such differences relate to and are reinforced by institutional logics (e.g., [26,27]; for recent overviews, see [14,28,29]; also note 1 in S1 Notes).

Although this (and related) work has been impressive, most scholars (explicitly or implicitly) have followed two understandings in using symbolic boundaries: First, scholars have mainly used the concept to analyze how people judge *others*. What has rarely been asked is whether and how symbolic boundaries might affect individuals’ ideas about *themselves*. An answer to this question might help to understand more fully how symbolic boundaries become effective in everyday life. A second limitation of existing work is that scholars have focused almost exclusively on how symbolic boundaries align with and thus reproduce social differences. Less studied has been the question of what happens when symbolic boundaries are violated. Yet attending more closely to such violations could help to understand the role of symbolic boundaries for social influence and thus social change.

In this paper, I develop a conceptual and empirical approach to symbolic boundaries that complements existing work by directly addressing these two limitations in order to advance an understanding of how symbolic boundaries might moderate social influence. First, I develop an understanding of how symbolic boundaries shape individuals’ decisions about themselves. Most behavior (or tastes or opinions) are not simply classified as “good” or “bad” in general; they are classified as good or bad for a certain type of person. Symbolic boundaries capture such cultural classifications. While these cultural classifications are essentially ascriptive, I argue that they also affect how people (selectively) attend to and take into account the behavior/opinions of others as people determine their *own* self-classifications and behavior. If people selectively attend to others who are on the same side of a salient symbolic boundary to evaluate their own behavior, symbolic boundaries might turn out to be important moderators of social influence.

Second, I focus on processes of boundary violation; that is, what happens if people express opinions or enact behavior that contravenes what is considered (in)appropriate for people like them. Specifically, I argue that observing such boundary violations might motivate (or at least authorize) people to change their own classifications and behavior. Boundary violations could thus lead to changes in what behavior, opinions and tastes are considered (in)appropriate for what kinds of people. If people selectively adjust their behavior based on violations made by others in their own symbolic groups, such a phenomenon might be key to understanding how symbolic boundaries stimulate and channel the direction of social change more generally.

Building on this, I then derive a model of social influence that takes the potential role of boundary violation into account. I test this model empirically using the case of drinking behavior among adolescents from different religious traditions. If the argument above is correct, an adolescent’s own drinking should be more influenced by the drinking of members of their own religious tradition. To investigate this possibility, I draw on data from Add Health, a nationally representative survey [30] and leverage a series of statistical techniques for observational data to arrive at the best possible estimates of the effect of symbolic boundary violation. I find that although the number of drinking peers has a small effect on drinking (as we would expect from standard peer influence models that do not consider the role of symbolic boundaries), exposure to boundary violations of one’s own symbolic group—having a same-religion friend who drinks—is a much stronger predictor of drinking behavior. This finding is consistent with the claim that symbolic boundaries moderate social influence. I conclude by discussing the implications of this model for cultural sociology more generally. Although not a perfect test, this first application helps us to establish the plausibility of the broader theoretical argument.

## Symbolic boundaries

A common and particularly influential way to understand symbolic boundaries is as widely shared demarcations between kinds of people, groups and things that are created, invoked, and negotiated in norms, cultural practices, and attitudes ([14], also [28,29]). If we focus on symbolic boundaries between kinds of people, symbolic boundaries mark what is considered (un)worthy, (dis)honorable, and (in)appropriate for members of particular groups (e.g., [31]). We can account for the logic of this understanding by conceptualizing symbolic boundaries as dual classifications of types of people on the one hand, and types of behavior/opinions on the other hand—*these* kinds of people do *these* kinds of things and *those* kinds of people do *those* kinds of things (for a formal account of this, see [32]).

Symbolic boundaries are not synonymous with social identifications. While social identifications are important for how people classify others into groups, symbolic boundaries consist in *how* (and only if) these group classifications are associated with expectations about behavior, opinions, and tastes considered appropriate or inappropriate for their members—in short, symbolic boundaries *are* the differential associations between group identifications *and* such expectations. Of course, to the extent that group identifications rest on ascribed rather than achieved characteristics and self-identifications, people may, in turn, also use knowledge about the behavior and opinions of others to aid classifying them into groups (related, see processes of “boundary work” as the construction and enactment of such expectations to invoke group differences). Nevertheless, keeping group identifications and expectations about the behavior, opinions and tastes appropriate for members of these groups conceptually separate is necessary for theorizing mechanisms that build on the interplay between the two.

From an individual’s perspective then, the dual classifications marked by symbolic boundaries define primarily one’s own group in relation to other groups [33]. They capture implicit and explicit understandings of what type of behavior is and is “not for the likes of us” (see [10]: 470ff., also [34]: 76ff.) and thus represent one basis of symbolic distinctions [10]. In negotiating these understandings, people tend to rationalize between-group differences by positively evaluating their own group’s typical behavior and/or negatively evaluating the typical behavior of outgroups (see [7,35]).

## Symbolic boundaries and the self

In using symbolic boundaries, scholars have largely followed two understandings: First, scholars have focused almost exclusively on how people classify and morally judge behavior and opinions in *others* (either of their own group or of other groups). Examples include studies of how individuals create, mobilize and negotiate “typical” differences among professionals and managers [25], the poor and the “deserving” [21], or among different types of members in social movements [36]. This emphasis on the ascriptive aspect of symbolic boundaries holds even for scholars attending to how people consider others’ views about one’s own symbolic boundary (e.g., [23]).

Although this is undoubtedly an important aspect, symbolic boundaries also play an important role for how people understand *themselves* and their *own* behavior. As widely shared schema (mental representation), symbolic boundaries entail self-classifications that bind together patterns of behavior, opinions and tastes and give them a moral significance [37]. As such, they both directly and indirectly influence how people think about themselves. On the one hand, they directly influence how people define and enact their identities as they provide widely shared and publicly accepted templates to draw on as they negotiate their identity [38,5,6].

On the other hand—and this has received less attention in the cultural literature—symbolic boundaries likely also indirectly influence people’s decisions about themselves. People can classify themselves (as they do others) based on a variety of characteristics. As markers of important distinctions, symbolic boundaries make certain classifications and related behavioral norms/expectations salient. They thus define which kind of people to attend to and compare oneself with as well as how to evaluate their behavior and opinions in determining one’s own behavior and opinions.

To give a (simple) example, for large parts of the Western world, crying to express sadness is considered acceptable in girls but shunned in boys. A symbolic boundary captures this dual-classification between types of people (boy/girl) and behavior (non-crying/crying), thus binding possible self-categorizations and behavioral norms/expectations—as a boy, you don’t cry. If salient, this symbolic boundary also affects how boys and girls selectively attend to and evaluate behavior in others as relevant for their own behavior. For example, because of symbolic boundaries, a boy may not regard girls’ frequency of crying around him as informative about how he ought to behave.

## Boundaries, reproduction, and change

Second, scholars trying to understand social differences have mainly focused on the role of symbolic boundaries for social reproduction. For example, Bourdieu and Passeron [26] argue that the educational system reproduces social inequality by evaluating children based on their familiarity with cultural forms of the dominant class, or Wimmer [39] describes how ethnic classifications essentially result from struggles over symbolic boundaries that are motivated by the institutional order, distribution of power, and political networks.

While understanding social reproduction is important, a sole focus on reproduction makes it also difficult to understand mechanisms related to social change as it encouraged scholars to focus nearly exclusively on conditions in which symbolic boundaries align with and reinforce social differences and institutional logics. We can do more to understand how symbolic boundaries shape social change by focusing on processes of *boundary violations*; that is, what happens if people engage in behavior or express opinions that are considered inappropriate for people like them. Although some scholars have attended to boundary violations, they have mainly theorized them as part of boundary work—that is, as episodes that are selective recalled and disapproved in public to confirm and thus maintain existing boundaries (e.g., scientists’ disapproval of “pseudo” and “deviant” scientists in [40,41]).

However, we can more fully theorize the role of boundary violations for social change. Boundary violations are not only opportunities to do “boundary work” required to maintain boundaries, but might also be important stimuli for cultural and social change. In particular, observing boundary violation might motivate people to adjust their own cultural classificatory systems of what behavior, opinions and tastes are considered (in)appropriate for what kinds of people. These adjustments might affect how people engage with others for whom shifts in understandings have occurred and, as they aggregate, might affect the symbolic boundary itself (see discussions of the private/public culture nexus in [42]; also [43–45]).

While we can expect such adjustments to have social consequences more generally, we can expect immediate changes in people’s behavior if symbolic violations concern their own classifications. We can do so for (at least) two reasons: First, as far as symbolic boundaries direct people’s attention towards others who belong to their own symbolic group and their behavior/opinions, people should also more likely notice and care more about boundary violations committed by these people than by others. Second, observing boundary violations that concern people’s own classifications should trigger changes in their understandings of what kind of behavior, opinions, and tastes are appropriate for themselves and therefore stimulate them to alter their own behavior, opinions, or tastes. For example, unlike girls’ frequency of crying (boundary compliance), observing that of boys around him (boundary violation) might motivate a boy to adjust his own classificatory system about what behavior is appropriate for boys; as a result, he might be more inclined to cry himself. (For a possible alternative if person classifications are malleable, see note 2 in S1 Notes.)

## Symbolic boundaries as moderators of social influence

If correct, the above argument implies that symbolic boundaries might be an important moderator of social influence and thus an important piece in the puzzle of understanding social change. In particular, it implies a mechanism for how symbolic boundaries might induce and channel social change. If salient symbolic boundaries define people’s selective attention and differential evaluation of (observed) behavior or (expressed) opinions in others, boundary violations might stimulate changes in their understandings of which kinds of behavior/opinions are acceptable for themselves, which motivate (or at least authorize) people to change their behavior/opinions.

Accordingly, we would expect that the strength of peer influence differs depending on whether a specific peer is part of one’s own symbolic group or not. In particular, if peers that are part of one’s own symbolic group engage in behavior that is considered inappropriate for members of that group this should have more of an influence on one’s own behavior than the sheer number of one’s peers that engage in such behavior. To a substantial degree, social change might thus be a function of the selective attention (and differential evaluation) induced by symbolic boundaries, especially as they are violated. (For the relation of this argument to reference group theory, see note 3 in S1 Notes.)

## The case of religion and adolescent drinking

A first step towards establishing the plausibility of this line of argument is to apply it to a concrete case and test whether the implied mechanism buys us anything beyond the standard model of social influence. An ideal case would be a symbolic boundary that combines distinct self-identifications with observable behavior that is susceptible to social influence. The case of religion and adolescents’ drinking behavior is such a case. Peer influence on drinking among adolescents are well established (e.g., [46–50]) and its negative consequences well-known, including academic impairments [51], increased health issues [52], involvement in severe traffic accidents [53,54], heightened levels of aggression [55] and suicide [56] (for a general overview, see [57]).

Religion provides a strong basis for self-identification in the US (e.g., [58–60]; also [61,62]; but [63]). We can therefore expect that classifications of others into religious traditions depend strongly on these self-identifications and thus offer a good candidate for studying the effects of symbolic boundaries associated with them. Moreover, research indicates that religious traditions mark symbolic boundaries that are salient with regard to adolescent alcohol use. For example, it is well established that religious affiliation and engagement are associated with lower levels of adolescent deviant behavior, including underage drinking [64–67]. These correlations are not incidental; they reflect a key way in which religious traditions use moral boundaries to create distinctive membership and identity (especially [68,60]). Accordingly, some scholars have used religiosity as an instrument for people’s alcohol consumption (e.g., [69–73]).

Applying the above line of argument to this case implies that having a drinking friend that also shares one’s religion—and thus observing deviant behavior violating the symbolic boundary of one’s own group—should predict drinking much more strongly than the simple number of drinking friends. Being exposed to a member of one’s own religion who drinks should encourage drinking by promoting an understanding that it is practically possible and socially acceptable for “people like me” to drink. A religious teenager who has friends of a different religious tradition (or no tradition) that drink may not be influenced by that fact because she does not see that behavior as relevant for her own decisions about drinking. She may infer that drinking is acceptable “for people like them” but not “for people like me.”

Based on this reasoning, I hypothesize that having at least one same-religion drinking friend will predict drinking frequency above and beyond the amount expected given the simple number of drinking friends (and the simple number of same-religion friends). Focusing on differences across religious traditions offers additional theoretical leverage: On the one hand, it allows me to explore whether the effect of boundary violation varies with the salience of the symbolic boundaries. Accordingly, I expect the effect to be stronger for religious traditions that uphold stricter norms against drinking such as Conservative/Evangelical Protestantism than those with more lenient prescriptions such as Mainline Protestantism (see [60,74,75]. On the other hand, because religious traditions differ in the emphasis they place on behavioral control, if we see the effect work for some traditions rather than others, this rules out that it is driven by religious affiliation per se rather than symbolic boundaries.

## Data

The National Longitudinal Study of Adolescent to Adult Health (AH) is ideal to test these predictions [30,76]). This nationally representative dataset contains measures of religious affiliation and respondent drinking as well as measures of network alters’ religion and drinking. Using named network alters is a much better way to define “peers” than using co-membership in school classes [48], schools [47,77], or neighborhoods [78] as more diffuse proxies (on this, see [79]: 129ff.). Because AH is a school-based sample, it also allows me to use school-level characteristics to model adolescents’ selection into boundary violating networks in order to identify peer influence with greater confidence. To the best of my knowledge, this is the only US dataset with such features.

I focus on AH’s wave I and II in-home interview data. In the 1994 to 1995 school year, 20,745 adolescents in grades 7 to 12 were interviewed at home. 7,106 adolescents in saturated schools were asked to list up to 5 of their closest male and female friends respectively. In 1996, 5,264 of these adolescents were re-interviewed and the same network information collected. The data allows matching respondents on friend nominations and thus to describe friends in terms of their own self-reports. The Institutional Review Board at Duke University approved this study.

## Measures

I construct the following measures (for descriptive statistics, see Table A in S1 Table):

### Drinking

This is a dummy variable capturing whether the respondents have drunk alcohol. In wave 1, respondents were first asked “Have you had a drink of beer, wine, or liquor—not just a sip or a taste of someone else’s drink—more than 2 or 3 times in your life?” In wave 2, they were asked, “Since [the last interview], have you had a drink of beer, wine, or liquor—not just a sip or a taste of someone else’s drink—more than 2 or 3 times?” Respondents could answer with “yes,” “no” or “don’t know.” I exclude respondents that did not know or refused to answer the question entirely.

### Religious tradition

AH asks detailed questions about respondents’ religious affiliations. I use a collapsed version of religious traditions [80].

### Number of drinking friends

I identify drinking friends based on their own self-reports using the same coding as above.

### Number of same-religion friends

This is the number of friends who share the respondent’s religious tradition/beliefs. I identify such friends based on a match between the respondent’s and his/her friends’ self-reported religious tradition.

### Boundary violation

I operationalize boundary violation concerning one’s own symbolic group by using an indicator variable set equal to 1 if the respondent has one or more friends who drink alcohol and share the respondents’ own religious tradition and 0 otherwise. That is, boundary violation captures the set overlap between drinking friends and same-religion friends (for a schematic display, see Appendix A in S1 Appendices).

### School-level proportions

I construct school-level proportions to capture the chance of meeting (1) someone who drinks alcohol, (2) someone who shares the respondent’s own religious tradition, and (3) someone who drinks alcohol and shares the respondent’s own religious tradition.

### Additional controls

This includes participants’ *gender*, *age*, frequency of *attending religious services* during the past year (ordinal with 4 levels: never / less than once a month / once a month or more / once a week or more) and a dummy for the interview *wave*.

## Analytical sample and strategy

Because of the nature of my hypothesis, I limit the sample as follows. I only analyze youth who identify with one of the three major religious traditions: Conservative/Evangelical Protestants, Mainline Protestants, and Catholics (80.7%). That is, I exclude non-religious adolescents since we have no way of knowing whether they belong to a group marked by a salient religious symbolic boundary (related, [81]) and religious traditions with insufficient cases for my subgroup analyses (for more details on this, see note 4 in S1 Notes). In constructing network characteristics for the remaining cases, I only consider friends for whom I know both their religious affiliation as well as their drinking behavior. I drop cases with missing data on drinking, religious tradition, network characteristics or any of the additional controls. This leaves us with an analytic sample of *N* = 4,510 adolescents (2,889 from wave I and 1,621 from wave II).

Ideally I would make use of the panel structure of the data to gain leverage over the question of causality by means of cross-lagged panel models or even fixed effects panel models to eliminate time constant unobserved heterogeneity at the individual level. However, I lack individuals who participated in both waves, switched drinking status in-between, and for whom I have sufficient network information. This is partly the case because participants’ network characteristics depend on answers from their friends. Given this, I chose to pool data from both waves and use dual equation models to get closer to an estimate of the effect of boundary violation on drinking.

My analytical strategy is as follows: Although the main dependent variable is dichotomous, I follow best practice in econometrics and use ordinary least squares (OLS) regression to establish the main effect (see [82]). This choice is especially sensible given the two equation approach described further below. Whereas the assumptions of OLS are well-known, there currently exists no reliable method to model the covariance between residuals in simultaneous logistic equation systems. Moreover, a two-stage substitution approach for logistic regressions produces biased and inconsistent estimates even if there is no unmeasured confounding. Potential alternatives include the two-stage residual inclusion approach, which is unbiased if no unmeasured confounding exists, but strongly biased otherwise (see [83]), and the double-logistic structural mean model (see [84]). Moreover, the use of OLS enables a direct comparison between estimates from the different single and dual equation systems shown below.

I begin by fitting a series of 6 regressions predicting drinking status. The key predictor is the boundary violation indicator—whether the respondent has at least one same-religion friend who drinks alcohol. I use robust standard errors, clustered within schools, to account for downward biases of estimated standard errors due to the “grouped” nature of the data (see [85]).

Models 1 to 4 take a standard full-sample regression approach and add increasing controls to isolate the effect of boundary violation. Model 1 is the standard model, with drinking status as a function of the number of same-religion and drinking friends alone. Model 2 includes only a dummy variable for boundary violation. Model 3 combines the two previous models. Model 4 adds controls, including an interaction term between the number of same-religion friends and the number of drinking friends. I include this interaction to rule out the possibility that it is the distribution of both sets of ties that is driving the effect rather than boundary violation per se. Moreover, Model 4 includes dummies for religious traditions.

Model 5 adds interaction terms between boundary violation and religious traditions to account for the possibly varying salience of religious denominations for drinking. Model 6 replaces the linear terms for the number of friends with indicators for each possible combination of same-religion and drinking friends in order to rule out that the effects of any combination of those distributions might drive the effect of boundary violation.

For each of the three different religious traditions, I then conduct a counterfactual analysis [86] to estimate the effect size of being embedded in a network that contains at least one boundary violation. I model drinking status among all adolescents who did not experience boundary violation, including indicators for the number of same-religion and drinking friends, gender, age, religious tradition and attendance. Because these regressions are limited to the sample of those who did not experience boundary violation, I had to assume that the effect of having 10 drinking or same-religion friends was the same as having 9. Based on the model estimates, I then predict the expected drinking status for those cases who were mathematically guaranteed to be in a boundary violating network and compare it to their observed drinking probability. Those are cases for which the sum of drinking friends and same-religion friends exceeds the maximum possible number of individual friends which implies that these two sets of ties necessarily need to overlap.

We need to exercise caution about interpreting estimates of these single equation models. The difficulty of inferring peer effects from observational data is well-known [87–91]. At least in parts, estimates of peer influence might reflect peer selection (reverse causality) or suffer from omitted variable bias. Especially with regard to the estimates for the effect of boundary violation we have reasons to doubt that this is the case (see note 5 in S1 Notes), but I cannot be certain. Different approaches to identify peer influence in observational data have been used (for overviews, see note 6 in S1 Notes and [92]). My last two models therefore apply an instrumental variable approach to arrive at the best possible estimate of the effect of boundary violation on drinking net of adolescents sorting into boundary violating networks. Of course, no single technique can establish the existence of a causal relationship beyond any doubt. But by using several different approaches with different assumptions, we can increase confidence in the robustness of the findings.

Instrumental variable (IV) methods are dual equation approaches that allow identifying the causal effect of an endogenous variable on the outcome in question. A valid instrument is a variable that meets two criteria: It needs to have a substantial direct impact on the outcome, and, net of controls, it needs to affect the outcome solely through its effect on the endogenous variable while being uncorrelated with unobserved covariates of the outcome. Whereas the first assumption can be tested, the second assumption can be supported but never fully validated by empirical results (for a succinct explanation, see [93]).

Accordingly, I conceptualize adolescents’ sorting into boundary violating networks as a matter of choice at the school level and use the chance to meet a boundary violating other at school as an instrument (for comparable IV approaches to eliminate selection biases, see [94]; also [95]). Everything else being equal, and net of the chances to meet drinking others and same-religion others per se, I would expect adolescents to be more likely to make boundary violating friends the higher the prevalence of those at school.

This choice of instrument rests on two assumptions. First, consistent with my argument, I assume that boundary violating others influence one’s own drinking solely if they breach into one’s friendship network. Results support this assumption; i.e. net of controls including the prevalence of drinking others and of same-religion others at school, the prevalence of same-religion drinking others at school shows no significant effect on drinking. Second, although this modelling strategy explicitly models peer selection, it implicitly assumes that school composition is exogenous to adolescents’ friendship choices. (Note, adolescents might well prefer schools with a higher/lower rate of pupils that share their own religion and/or a higher/lower rate of pupils who drink; the specific assumption to be met is only that there is no sorting into schools based on the prevalence of boundary violating others at school *beyond* possible sorting based on the prevalence of drinking or same-religion others at school.)

To implement the IV approach, I thus model drinking in two stages. First, I regress boundary violation on the prevalence of drinking others, same-religion others and boundary violating others at school as well as all previous controls. Second, I regress drinking on the estimated boundary violation from the first stage (Model IV). Finally, Model IV (FE) adds fixed effects for schools, limiting the estimation to within-school variance and thus eliminating sources of bias that are constant at the school level.

The IV approach is particularly well-suited to estimate effects like these. This is because the IV approach yields an estimate for the average effect of boundary violation on drinking behavior for all those who “chose” to have boundary violating friends because of the availability of such friends at their school, regardless of the effects for those who would have self-selected into friendships with boundary violating others for reasons unrelated to the availability of boundary violating others and that might also be related to their drinking behavior (for an expression of this in the logic of treatment effect estimation, see note 7 in S1 Notes). Although this might not be ideal from a public policy perspective, the IV approach—if its assumptions are justified—thus allows us to get closer to an estimate of the influence effect of boundary violation net of that of self-selection. (See Appendix B in S1 Appendices for a simulation demonstrating this point.) Of course, this is *not* decisive proof; however, it helps provide additional plausibility for a possible causal interpretation.

## Results

My results support the hypothesis that the boundary violation model of peer influence is a better fit to the data than the standard model alone. In all models, respondents who have at least one same-religion drinking friend are predicted to be more likely to drink than would be expected by the number of same-religion and drinking friends alone.

Table 1 shows the main estimates for the single equation Models 1 to 6 (for complete estimates, see Table B in S1 Table).

**Table 1:** Linear probability models of drinking.

	(1)		(2)		(3)		(4)		(5)		(6)	
	AddVector		Naive		Combined		Controls I		Controls II		+JointDist	
Same-religion friends	-0.04	***			-0.06	***	-0.07	***	-0.07	***		
	(3.82)				(5.78)		(11.37)		(11.17)			
Drinking friends	0.07	***			0.06	***	0.04	***	0.04	***		
	(8.67)				(10.45)		(5.81)		(5.83)			
Boundary violation (BV)			0.13	***	0.14	***	0.13	***	0.17	***	0.19	***
			(8.36)		(9.80)		(9.05)		(9.55)		(6.08)	
R: Conservative protestant							*ref*.		*ref*.		*ref*.	
R: Mainline protestant							-0.00		0.05	*	0.04	*
							(0.02)		(2.20)		(1.98)	
R: Catholic							0.05	***	0.08	***	0.08	***
							(3.88)		(3.81)		(4.26)	
BV * Conservative protestant									*ref*.		*ref*.	
BV * Mainline protestant									-0.16	**	-0.11	
									(3.07)		(1.77)	
BV * Catholic									-0.06	*	-0.07	*
									(2.00)		(2.60)	
Joint Dist. Dummies	No		No		No		No		No		Yes	
*N*	4510		4510		4510		4510		4510		4510	

**Note:** Sample is limited to religious adolescents who specified belonging to Conservative Protestant, Mainline Protestant, or Catholic religious traditions. All models control for interview wave. Models 4 to 6 also control for sex, age, religious tradition, religious attendance, the product of the number of same-religion friends and the number of drinking friends. Models 5 and 6 control for the interaction between religious traditions and boundary violation status. Model 6 includes indicator variables for all empirical combinations of the number of same-religion friends and the number of drinking friends. Absolute *z* statistics in parentheses; robust standard errors, clustered within schools.

* p < 0.05,

** p < 0.01,

*** p < 0.001 (two-tailed tests).

Model 1 is a simple model that estimates drinking as a function of the number of same-religion friends and the number of drinking friends. As expected, the number of same-religion friends is negatively associated with drinking and the number of drinking friends is positively associated with drinking. Each additional same-religion friend reduces the expected drinking probability by 4 percentage points and each additional drinking friend increases the expected drinking probability by 7 percentage points.

Model 2 includes only a dummy variable for boundary violation—whether the respondent has at least one friend who is of his or her same religious tradition *and* who drinks alcohol. Respondents with boundary violating networks are 13 percentage points more likely to drink.

Model 3 combines the standard model with the boundary violation indicator. While the additive effect of drinking friends decreases, the boundary violation coefficient increases. In this model, respondents with boundary violating networks are 14 percentage points more likely to drink, even controlling for the number of same-religion friends and the number of drinking friends.

Model 4 adds controls to Model 3, including an interaction term between the number of same-religion friends and the number of drinking friends and controls for religious tradition. I include the interaction term to rule out the possibility that it is not the theorized overlap of the drinking and same-religion sets but rather the distribution of both sets of ties that is driving the boundary violation effect. The addition of the controls does little to the boundary violation coefficient while the effect of the number of drinking friends drops even further. Moreover, consistent with previous findings, I find that Catholics are more likely to drink than Conservative/Evangelical or Mainline Protestants.

Model 5 accounts for difference in the boundary violation effect that corresponds to the varying strength of the symbolic boundary by religious tradition. The results show that boundary violation effect is strongest for Conservative/Evangelical Protestants (17 percentage points), followed by Catholics (11 percentage points) and barely existing among Mainline Protestants (1 percentage point). This corresponds to known variations in the salience of corresponding religious beliefs with regard to drinking [68,74,96] and rules out that the effect of boundary violation is due to religious affiliation per se.

Model 6 includes dummies for each of the observed combinations between number of same-religion friends and number of drinking-religious friends. Results remain nearly unchanged, except that the interaction between boundary violation and being a Mainline Protestant drops below significance, which is likely due to this group being the smallest (for estimates of further variants of these models, see note 8 in S1 Notes).

Finally, I conduct counterfactual analyses (discussed above) to estimate the effect of experiencing a boundary violating tie. For each of the three religious traditions, I model drinking experience among all adolescents who did not experience boundary violation, including dummies for the number of same-religion and drinking friends, gender, age, religious tradition and attendance. Based on the model estimates, I then predict the expected drinking probability for cases outside of the area of common support. Difference in probabilities tests show that, for Conservative/Evangelical Protestants, the proportion of adolescents who drank among those who experienced boundary violation is 24 percentage points higher than we would have expected if they had not, and for Mainline Protestants 12 percentage points and for Catholics 10 percentage points (see Table 2).

**Table 2:** Tests of proportions of observed against predicted drinking for cases with guaranteed boundary violation by religious tradition.

	*N*	Proportion	*SD*	Difference	*p*-value (two-sided) (one-sided)	
Conservative protestant						
Proportion drinking (observed)	831	0.62	0.49			
Proportion drinking (predicted)		0.38	0.17	0.24	0.000	0.000
Mainline protestant						
Proportion drinking (observed)	122	0.53	0.50			
Proportion drinking (predicted)	122	0.41	0.17	0.12	0.051	0.026
Catholic						
Proportion drinking (observed)	1008	0.65	0.48			
Proportion drinking (predicted)	1008	0.55	0.21	0.10	0.000	0.000

**Note:** Effects of 9 and 10 same-religion/drinking friends assumed to be equal.

To this point, the results are strongly consistent with the hypothesis that symbolic boundary violation has a qualitative effect on drinking among adolescents. Moreover, the best controlled results (Models 5 and 6) are consistent with a theoretical model of social influence that argues that drinking friends matter mainly when they are members of one’s own religious group. However, we should be cautious of premature interpretations. These estimates might be biased to the extent that having boundary violating friendships itself is a matter of choice or determined by unobserved variables. In the extreme, one might argue that the estimated effect of boundary violation on drinking merely reflects the tendency of drinking adolescents to prefer friends who drink *and* share their own religious tradition *beyond* their preferences for drinking friends and/or same-religion friends per se.

I tackle this issue by using an IV approach (described above) and instrument boundary violation by the chance to meet a potential boundary violating friend at school. In a first equation, I regress boundary violation on the prevalence of drinking others, same-religion others and boundary violating others at school as well as all previous controls (see Table C in S1 Table). Results show that the prevalence of boundary violating others at school has a statistically significant and strong effect on boundary violation, alleviating concerns about weak instrument biases (on instrument validity and sensitivity, see [97]).

Second, I regress drinking on the estimated boundary violation from the first equation (see Table 3, Model IV). Results show that respondents with boundary violating networks are 31 percentage points more likely to drink. This raises strong doubts for the argument that the found peer effects merely reflect self-selection processes; if anything, results suggests that possible sorting into boundary violating networks had biased downwards the estimates of boundary violation on drinking status in the single equation models. Moreover, the effect of number of drinking friends becomes indistinguishable from 0 while the other estimates remain similar to the full-control estimates in Models 5 and 6.

**Table 3:** Two equations linear probability model of drinking and of boundary violation instrumented by the school proportion of same-religion drinking friends.

	IV				IV (FE)			
	BV		Drinking		BV		Drinking	
Same-religion friends	0.19	***	-0.08	***	0.19	***	-0.09	***
	(7.56	)	(4.90)		(7.40)		(4.02	)
Drinking friends	0.13	***	0.01		0.14	***	0.00	
	(5.08)		(0.92)		(4.83)		(0.27)	
Boundary violation (BV)			0.31	***			0.34	**
			(3.92)				(3.06)	
Same-religion * drinking friends	-0.02	***	0.01		-0.02	***	0.01	
	(3.54)		(2.74)		(3.56)		(2.57)	
Female	0.02		-0.02		0.02		-0.02	
	(1.74)		(1.81)		(1.70)		(1.81)	
Age	0.01		0.03	***	0.01		0.03	***
	(1.63)		(5.08)		(1.80)		(4.44)	
Religious attendance	0.00		-0.04	***	0.00		-0.04	***
	(0.91)		(5.59)		(0.78)		(5.56)	
R: Conservative protestant	*ref*.		*ref*.		*ref*.		*ref*.	
R: Mainline protestant	-0.13	***	0.07	**	-0.14	***	0.07	**
	(4.18)		(3.03)		(4.55)		(2.30)	
R: Catholic	0.07		0.06	**	0.04		0.06	**
	(1.56)		(2.69)		(0.81)		(2.38)	
BV * Conservative protestant			*ref*.				*ref*.	
BV * Mainline protestant			-0.13	**			-0.13	**
			(2.93)				(2.91)	
BV * Catholic			-0.05				-0.04	
			(1.77)				(1.43)	
Interview wave	-0.04	**	-0.05	***	- 0.03	**	-0.05	***
	(2.63)		(4.28)		(2.34)		(4.50)	
School prop. drinking friends	- 0.38	***	0.34	***	-0.29	**	0.31	***
	(4.26)		(5.03)		(3.25)		(3.55)	
School prop. same-religion friends	-0.56	***	0.03		-0.50	***	0.01	
	(6.33)		(0.69)		(3.70)		(0.25)	
School prop. same-religion drinking friends	1.62	***			1.43	***		
	(9.84)				(5.58)			
School prop. same-religion drinking friends * Conservative protestant	*ref*.				*ref*.			
School prop. same-religion drinking friends *	0.41				0.35			
Mainline protestant	(1.61)				(1.52)			
School prop. same-religion drinking friends *	-0.26				-0.12			
Catholic	(1.40)				(0.57)			
Intercept	0.07		-0.08		-0.03		-0.11	
	(0.69)		0.70		0.27		0.71	
*N*	4510				4510			

**Note**: Sample is limited to religious adolescents who specified their religious tradition and to cases with an analytical school size larger than the number of friends. Model IV (FE) includes fixed effects for schools. Absolute z statistics in parentheses; robust standard errors, clustered within schools.

* p < 0.05,

** p < 0.01,

*** p < 0.001 (two-tailed tests).

Finally, I include school fixed effects to control for possible context effects that apply equally to all students in each school (see Table 3, Model IV (FE); for school-level intra-cluster correlation coefficients, see Table D in S1 Table). The effect of boundary violation on drinking gets even stronger while the other effects remain essentially unchanged (for a further check, see note 9 in S1 Notes).

**Fig 1:**
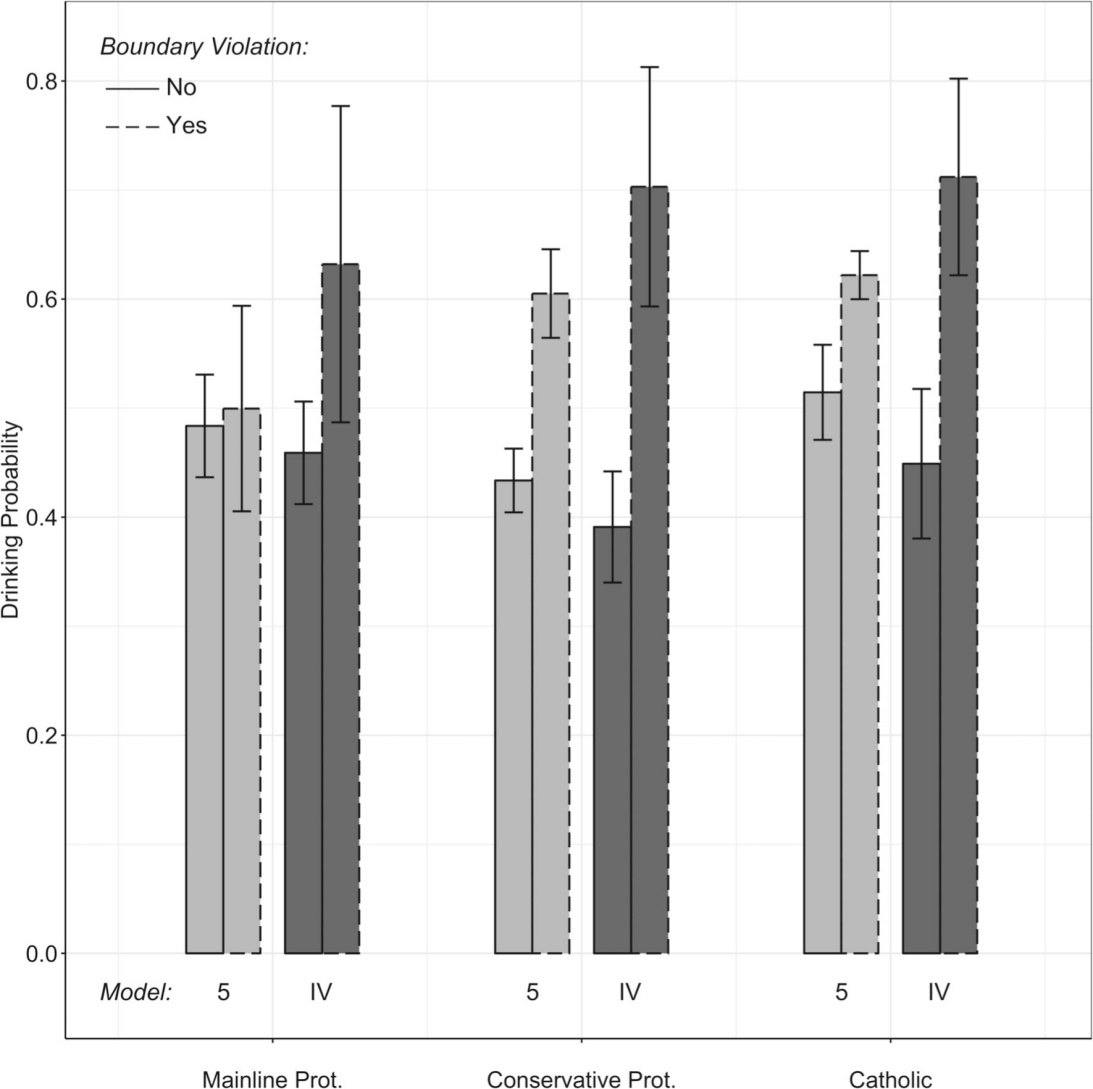
Point estimates and 95% confidence intervals for the predicted drinking probability for respondents with and without a boundary violating tie by religious tradition. Reported are predicted probabilities for cases with 1 drinking friend, 1 religious friend, and mean values on all other variables based on estimates from the full control Model 5, Table 1 and the instrumental variable Model IV, Table 3. Boundary violation is defined as having a same-religion friend who drinks.

Fig 1 displays predicted drinking probabilities for the adolescents with and without a boundary violating tie for each of the three religious traditions based on estimates from the full-control Model 5 (Table 1) and instrumental variable Model IV (Table 3). In all models, respondents who have at least one same-religion drinking friend are predicted to more likely drink than would be expected by the number of same-religion friends and drinking friends alone.

Moreover, a sensitivity analysis following Frank [98] shows that, to invalidate these results for the full-control Model 5 (Table 1) and the instrumental variable Model IV (Table 3), a potential confounder would have to be correlated with observing boundary violation and drinking behavior to an extent that corresponds to replacing, respectively, at least 79.5% and 49.7% of all cases in the sample with cases for which there is no boundary violation effect. In sum, results suggest that the effect of boundary violation on drinking behavior is generally strong, and while (as in any observational data) it is ultimately impossible to rule out unobserved selection features, the literature does not suggest additional factors that could have the necessary association to invalidate the found estimates net of the included controls.

## Discussion and conclusion

In this paper, I have investigated how symbolic boundaries shape social influence and behavior by addressing two limitations of previous research. First, I have developed an understanding of how symbolic boundaries shape individuals’ ideas and decisions about themselves, not only others. While being essentially ascriptive, I have argued that these classificatory systems also affect how people (selectively) attend to and evaluate the behavior/opinions of others as they determine their *own* self-classifications and behavior.

Second, beyond their role in social reproduction, I have argued that symbolic boundaries might also shape cultural and social change and that we can understand this more fully by focusing on boundary violations—that is, what happens if people enact behavior that contravenes what is considered (in)appropriate for people like them. In particular, observing members of one’s *own* symbolic group commit such violations might motivate (or at least authorize) people to adjust their own classifications and behavior.

To demonstrate the utility of this line of argument, I tested this mechanism with regard to the case of religion and adolescents’ drinking behavior. Results yielded strong support for the hypothesis that models that take boundary violations into account are a better fit to the data than the standard model of social influence. In all models, respondents who have at least one same-religion drinking friend (i.e., experience boundary violation with regard to their own symbolic group) are predicted to drink more than would be expected by the number of same-religion and drinking friends alone (or their linear interaction). The strict test of only comparing respondents with exactly the same numbers of co-religious and drinking friends shows an even stronger effect.

Furthermore, results from an instrumental variable approach are at least consistent with the assertion that this peer effect is not an artifact of sorting into boundary violating networks. In this regard, my findings are similar to that of others who find that peer effects persist even after accounting for social selection [47,48,77] and that the predicted marginal peer influence effect increases once we account for social selection [48]. Moreover, based on these results, there appears to be no direct influence of the simple number of drinking friends on respondent drinking; only whether or not the respondent has a same-religion drinking friend predicts their own drinking behavior.

This study is of course limited in specific ways. First, I control for the effect of drinking friends and/or same-religious friends as well as account for the possibility that respondents select into boundary violating friendships themselves. Nevertheless, my modelling strategy implicitly treats the prevalence of boundary violating others at school *beyond* the prevalence of drinking or same-religion others at school as exogenous. This means that my approach might over- or underestimate the effect of boundary violation on drinking in so far as factors exist that influence *(i)* drinking behavior *and (ii)* the prevalence of boundary violating others at school beyond the prevalence of drinkers at school or the religious makeup of the school population.

Second, it rests on the assumption that at least some religious traditions form symbolic groups that are salient with regard to drinking in the US. This is likely the case (see [60,64,68,74,75,96]), but I cannot say for sure.

Third, although adding nuance, my sub-group analysis does not attend to possible differences within broad religious traditions in the US. For example, based on more fine-grained data, future research could explore differences between more or less liberal congregations.

Finally, it is possible that people experiencing boundary violations not only adjust their attitude towards drinking but also symbolically re-classify friends or even break ties (related, see practices of “boundary framing” in social movement theories in [99,36] and more generally [100,101]; further also note 10 in S1 Notes). The above estimates of the effect of boundary violation on drinking might still be conflated with such effects. In particular, observing a friend of one’s own religious tradition who drinks might foster doubts about his religious seriousness or even motivate denying that he belongs to that tradition. To the extent that such a friend is therefore not perceived as violating the symbolic boundary, my analysis above had overestimated the effect of boundary violation on drinking. In the extreme case, this experience might even motivate breaking off the friendship, in which case I would not even observe such relationships in the data (see [102]). Unfortunately, I cannot investigate these tendencies in my data (related, note 11 in S1 Notes).

This study should only be understood as a first, lose test of my broader theoretical argument but one that helps us to establish the plausibility of the implied mechanism. Much more conceptual and empirical work is needed before we can be sure of it. Conceptually, we need to specify the types of symbolic boundaries the mechanism applies to and identify boundary conditions beyond group membership. Empirically, we need to test the mechanism in many other situations, using alternative and more fine-grained measurements of group involvement and identification, and account for how the salience of symbolic boundaries varies by context.

Despite these potential limitations my findings have important implications for understanding social influence and cultural sociology more generally. For understanding social influence, they imply that scholars would benefit from taking the role of cultural classifications as sources of heterogeneous peer effects into account. In modelling peer influence, scholars generally assume (for simplicity) that agents are indifferent about which peers enact a behavior or hold an opinion (see [92]). The results here demonstrate that some peers matter more than others for influencing behavior because not all are members of the same symbolic groups. Models of network influence can therefore be improved by taking symbolic categorizations into account.

For cultural sociology (and social theory more generally), my findings offer both methodological and theoretical implications. Methodologically, they show that to unravel the working of culture in everyday life, approaches attuned to its classificatory and thus often qualitative logic can be extremely helpful. Accounting for the joint distribution between people and behavior in a classificatory fashion was essential to successfully detect the crucial role that symbolic boundaries play in this particular case. While others have studied heterogeneous peer effects in the past by means of linear interaction effects (e.g., [103]) or subgroup analysis (e.g., [50,104]), we need more suitable network data and modeling strategies that go beyond modeling influence as a quantitative force or threshold (see [105]).

Theoretically, my findings imply that, beyond their role in social reproduction, symbolic boundaries also need to be understood as potential catalysts for socio-cultural change. As sources of selective attention to others and differential evaluation of their behavior, opinions, and tastes, symbolic boundaries function as moderators of social influence. Especially through their violation, symbolic boundaries might thus initiate and channel cultural and social change.

## Supporting information

S1 Notes (PDF)

S1 Tables (PDF)

S1 Appendices (PDF)
