# Supplementary material for: Boundary violations and adolescent drinking: Observational evidence that symbolic boundaries moderate social influence
Source: PLoS One. 2019 Nov 5;14(11):e0224185. doi: 10.1371/journal.pone.0224185 (PMC6830941; doi:10.1371/journal.pone.0224185)
Supplement: S1 Notes — (PDF) [file pone.0224185.s001.pdf]

S1 Notes.

1 Many other important works beyond these exemplary studies could and should be mentioned here if space would permit; unfortunately, even review articles struggle in surveying this field in its entirety (see [1]).

2 If person classifications are malleable, people might also reclassify themselves and/or others. For example, they might question whether someone qualifies as a “true” member of their group or they might renounce their membership themselves. Although interesting in itself, in this paper I focus on the question of whether and how people might change their own behavior as a result of observing boundary violations instead and thus what can be addressed using observable behaviors and reported self-classifications.

3 Similar to the line of argument proposed here, also reference group theory [2] makes statements about self-identification and social comparison. Unlike reference group theory, however, symbolic boundaries also conceptualize what kinds of behavior/opinions are considered salient characteristics of these groups as well as how they are evaluated, thus implying both the content of social influence and the direction of change it stimulates. Not surprisingly, a known difficulty for reference group theory has been whether to define in- and out-group classifications based on either categorical membership or social interaction (or both) (see [2]: 287); in contrast, the concept of symbolic boundaries keeps these two forms of social associations analytically separate, which allows to specify the above model of peer influence.

4 I also conducted the analyses for an extended sample that includes Jewish, Mormon, and Hindu/Buddhist/Islamic traditions ($N = 37$, 47, and 37 respectively). Given that results for such small subgroups should only be interpreted with great caution, and given that not all analyses could be conducted for these subgroups because of their sizes, I only indicate found tendencies in supplementary notes.

5 Previous studies on homophily in networks found a decreasing effect of similarity across multiple characteristics (e.g. [3]). Applied to this case, this would even imply a negative association between boundary violation and drinking if adolescents would select into friendships based on the perceived boundary violation of others net of the effects of drinking friends and same-religion friends.

6 This includes the use of fixed effects models [4], instrumental variables and structural equations [5], lagged peer group characteristics [6], use of partners' friendships [7] randomly assigned roommates [8,9], and quasi-experimental designs [10]; for an overview, see [11].

7 Expressed in the logic of treatment effect estimation: If boundary violation is considered a treatment, the IV approach taken here allows estimating the average treatment effect among the compliers net of that among non-compliers (and always takers).

8 I fit three further variants of the models presented in the main Table 1: First, I fit Models 1 to 6 to an extended sample that includes adolescents identifying as Jewish, Mormon, and Hindu/Buddhist/Islamic (N = 37, 47, and 37 respectively). I do not report on them in detail here. As we would expect from the argument, the effect of symbolic boundary violation is strong among Mormons who uphold very strict norms against drinking. Estimates for Jewish and Hindu/Buddhist/Islamic traditions point in the expected directions but are non-significant, likely due to a lack of statistical power. Unfortunately, due to a lack of cases, the counterfactual and IV analysis could not be conducted for these subgroups.

Second, including dummies for the interview wave accounts for some but not all shortcomings of pooling data across waves 1 and 2. For example, due to differences in the question wording, the measurement of drinking status and thus also of boundary violation captures different time periods in both waves. Because of this, the effect of boundary violation on drinking could appear as more or less pronounced in the two waves. To account for this possibility, I fit Models 2 to 6 including an interaction term for boundary violation and interview wave. Results (not shown) reveal no significant effect for this interaction term.

Finally, given possible limitations of using ordinary least square regression in predicting a binary outcome, I fitted Models 1 to 6 using logistic regression instead (see Appendix C in S1 Appendices); the result pattern is substantially the same as the one reported in the main Table 1.

9 I also tried instrumenting boundary violation with the interaction between the prevalence of the drinking others and the prevalence of same-religion others at school; results (not shown) are similar.

10 For solutions to structurally analogous situations, see also rituals of "status degradations" [12].

11 One way to account for the first possibility is to use data in which respondents themselves describe both friends' drinking status and religious tradition; accounting for the second possibility requires richer longitudinal data to account for the discontinuation of relationships. Mixed-methods studies might also be particularly helpful to reconstruct the experience of such events [13].

References in S1 Notes.

[1] Lamont M, Pendergrass S, Pachucki MC. Symbolic boundaries. In: J Wright, editor. International Encyclopedia of Social and Behavioral Sciences. Oxford: Elsevier; 2015. p. 850-55.

[2] Merton RK. Social Theory and Social Structure. Simon and Schuster. 1968.

[3] Block P, Grund T. Multidimensional homophily in friendship networks. Network Science. 2014 Aug;2(2):189-212.

[4] Lundborg P. Having the wrong friends? Peer effects in adolescent substance use. Journal of Health Economics. 2006;25(2):214-33.

[5] Norton EC, Lindrooth RC, Ennett ST. Controlling for the endogeneity of peer substance use on adolescent alcohol and tobacco use. Health Economics. 1998;7(5):439-53.

[6] Clark AE, Lohéac Y. "It wasn't me, it was them!" Social influence in risky behavior by adolescents. Journal of Health Economics . 2007;26(4):763-84.

[7] Kreager DA, Haynie DL. Dangerous liaisons? Dating and drinking diffusion in adolescent peer networks. American Sociological Review . 2011;76(5):737-763.

[8] Duncan GJ, Boisjoly J, Kremer M, Levy DM, Eccles J. Peer effects in drug use and sex among college students. Journal of Abnormal Child Psychology. 2005;33(3):375-85.

[9] Eisenberg D, Golberstein E, Whitlock JL. Peer effects on risky behaviors: New evidence from college roommate assignments. Journal of Health Economics 33. 2014:126-38.

[10] Guo G, Li Y, Owen C, Wang H, Duncan GJ. A natural experiment of peer influences on youth alcohol use. Social Science Research. 2015;52:193-207.

[11] Mouw T. Estimating the causal effect of social capital: A review of recent research. Annual Review of Sociology. 2006;32:79-102.

[12] Garfinkel H. Conditions of successful degradation ceremonies. American Journal of Sociology. 1956; 61(5):420-424.

[13] Small ML. How to conduct a mixed methods study: Recent trends in a rapidly growing literature. Annual Review of Sociology. 2011;37:57-86.
