# Supplementary material for: Boundary violations and adolescent drinking: Observational evidence that symbolic boundaries moderate social influence
Source: PLoS One. 2019 Nov 5;14(11):e0224185. doi: 10.1371/journal.pone.0224185 (PMC6830941; doi:10.1371/journal.pone.0224185)
Supplement: S1 Tables — (PDF) [file pone.0224185.s002.pdf]

S1 Tables.

I present four supporting tables: Table A shows sample descriptives for the main variables. Table B corresponds to Table 1 in the main text and shows the complete estimates for models 1 to 6. Table C shows the linear probability model of boundary violation (not instrumented / instrumented). Table D shows the school-level intra-cluster correlation coefficients.

Table A. Means and standard deviations of dependent and independent variables.

	Mean	SD	Min	Max
	Wave I (N = 2889)			
Drinking Status	0.59	0.49	0	1
Drinking friends	1.85	1.71	0	10
Same-religion friends	1.50	1.53	0	9
Boundary violation	0.54	0.50	0	1
Female	0.50	0.50	0	1
Age	16.53	1.55	12.08	20.92
Religious attendance	2.02	1.05	0	3
R: Conservative protestant	0.42	0.49	0	1
R: Mainline protestant	0.15	0.35	0	1
R: Catholic	0.43	0.50	0	1
School prop. drinking friends	0.59	0.14	0	1
School prop. same-religion friends	0.40	0.20	0	1
School prop. same-religion drinking friends	0.23	0.12	0	1
	Wave II (N = 1621)			
Drinking Status	0.47	0.50	0	1
Drinking friends	1.47	1.46	0	9
Same-religion friends	1.44	1.46	0	10
Boundary violation	0.43	0.49	0	1
Female	0.51	0.50	0	1
Age	16.99	1.48	13.34	21.58
Religious attendance	2.11	1.05	0	3
R: Conservative protestant	0.49	0.50	0	1
R: Mainline protestant	0.13	0.33	0	1
R: Catholic	0.38	0.49	0	1
School prop. drinking friends	0.48	0.14	0.05	0.67
School prop. same-religion friends	0.42	0.21	0	0.98
School prop. same-religion drinking friends	0.18	0.08	0	0.43

Sample limited to cases with school size larger than number of friends.

Table B. Complete linear probability models of drinking.

	(1) AddVector	(2) Naive	(3) Combined	(4) Controls I	(5) Controls II	(6) +JointDist
Same-religion friends	–0.04***		–0.06***	–0.07***	–0.07***	
	(3.82)		(5.78)	(11.37)	(11.17)	
Drinking friends	0.07***		0.06***	0.04***	0.04***	
	(8.67)		(10.45)	(5.81)	(5.83)	
Boundary violation (BV)		0.13***	0.14***	0.13***	0.17***	0.19***
		(8.36)	(9.80)	(9.05)	(9.55)	(6.08)
Same-religion * drinking friends				0.01***	0.01***	
				(4.36)	(3.93)	
Female				–0.02	–0.02	–0.02
				(1.57)	(1.56)	(1.65)
Age				0.04***	0.04***	0.04***
				(5.21)	(5.22)	(5.18)
Religious attendance				–0.05***	–0.05***	–0.05***
				(5.65)	(5.62)	(6.10)
R: Conservative protestant				*ref.*	*ref.*	*ref.*
R: Mainline protestant				–0.00	0.05*	0.04*
				(0.02)	(2.20)	(1.98)
R: Catholic				0.05***	0.08***	0.08***
				(3.88)	(3.81)	(4.26)
BV * Conservative protestant					*ref.*	*ref.*
BV * Mainline protestant					–0.16**	–0.11
					(3.07)	(1.77)
BV * Catholic					–0.06*	–0.07*
					(2.00)	(2.60)
Interview wave	–0.10***	–0.11***	–0.09***	–0.10***	–0.10***	–0.10***
	(3.98)	(4.27)	(3.74)	(5.31)	(5.24)	(5.26)
Intercept	0.62***	0.63***	0.59***	0.07	0.03	0.00
	(23.20)	(30.10)	(22.98)	(0.58)	(0.25)	(0.01)
Joint Dist. Dummies	No	No	No	No	No	Yes
N	4510	4510	4510	4510	4510	4510

Note: Sample is limited to religious adolescents who specified belonging to Conservative Protestant, Mainline Protestant, or Catholic religious traditions. All models control for interview wave. Models 4 to 6 also control for sex, age, religious tradition, religious attendance, the product of the number of same-religion friends and the number of drinking friends. Models 5 and 6 control for the interaction between religious traditions and boundary violation status. Model 6 includes indicator variables for all empirical combinations of the number of same-religion friends and the number of drinking friends. Absolute z statistics in parentheses; robust standard errors, clustered within schools.

* $p < 0.05$, ** $p < 0.01$, *** $p < 0.001$ (two-tailed tests).

Table C. Linear probability model of boundary violation.

	(1) Boundary Violation (not instrumented)	(2) Boundary Violation (instrumented)
Same-religion friends	0.18***	0.19***
	(6.63)	(7.57)
Drinking friends	0.12***	0.13***
	(4.14)	(5.09)
Same-religion*drinking friends	–0.02*	–0.02***
	(2.24)	(3.55)
Female	0.02	0.02
	(1.72)	(1.75)
Age	0.01	0.01
	(0.84)	(1.56)
Religious attendance	0.01	0.00
	(1.16)	(0.90)
R: Conservative protestant	*ref.*	*ref.*
R: Mainline protestant	–0.10***	–0.13***
	(3.63)	(4.18)
R: Catholic	0.06*	0.06
	(2.18)	(1.24)
Interview wave	–0.03	–0.04**
	(1.47)	(2.63)
Sc. prop. drinking friends	0.41**	–0.39***
	(2.97)	(4.30)
Sc. prop. same-religion friends	0.18*	–0.56***
	(2.07)	(6.38)
Sc. prop. same-religion drinking friends * Conservative Protestant		*ref.*
Sc. prop. same-religion drinking friends * Mainline Protestant		0.39
		(1.57)
Sc. prop. same-religion drinking friends * Catholic		–0.21
		(1.11)
Sc. prop. same-religion drinking friends		1.61***
		(9.82)
N	4510	4510
Chi^2		50718.15
df (Chi^2, 2-sided)		3.00
p (Chi^2, 2-sided)		0.00

Note: Sample is limited to religious adolescents who specified their religious tradition and to cases with an analytical school size larger then number of friends. Two-sided chi-squared test of difference in log-likelihood between excluding (Model 1) and including (Model 2) the instrument (school proportion of same-religion drinking friends) and necessary interaction controls (school proportion of same-religion drinking friends * Mainline Protestant / Catholic) reported. Absolute z statistics in parentheses; robust standard errors, clustered within schools.

* $p < 0.05$, ** $p < 0.01$, *** $p < 0.001$ (two-tailed tests).

Table D. School-level intra-cluster correlation coefficients.

	ρ
Drinking status	0.102
Drinking friends	0.101
Same-religion friends	0.140
Same-religion*drinking friends	0.045
Boundary violation	0.055
Female	0.009
Age	0.511
Religious attendance	0.146
R: Conservative protestant	0.220
R: Mainline protestant	0.224
R: Catholic	0.212
School prop. drinking friends	0.928
School prop. same-religion friends	0.694
School prop. same-religion drinking friends	0.751
N	4510

Sample limited to cases with school size larger than number of friends.
