# Supplementary material for: Boundary violations and adolescent drinking: Observational evidence that symbolic boundaries moderate social influence
Source: PLoS One. 2019 Nov 5;14(11):e0224185. doi: 10.1371/journal.pone.0224185 (PMC6830941; doi:10.1371/journal.pone.0224185)
Supplement: S1 Appendices — (PDF) [file pone.0224185.s003.pdf]

S1 Appendices.

I provide three supporting appendices referred to in the main text.

Appendix A: Operationalizing boundary violation

Boundary violation accounts for the set overlap between drinking friends and same-religion friends. Consider, for example, the two situations depicted in Fig A1. In both situation ego has two drinking friends and two same-religion friends but only in situation 2 these two sets of ties overlap and ego experiences boundary violation.

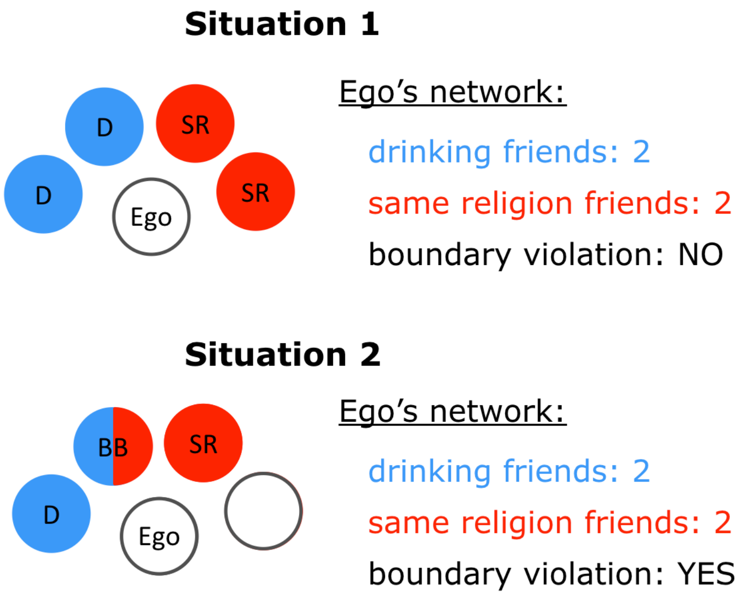

Fig A1. Schematic display of boundary violating network

Appendix B: Simulation — IV approach to estimate Local Average Treatment Effect (LATE) on the compliers.

The goal of the above analysis is to estimate a social influence effect even though the data does not allow for causal claims and it is impossible to collect data that could. In particular, I needed to estimate the effect of boundary violation on drinking behavior in observational data. This involves addressing the methodological problem of ensuring that estimates of this social influence effect are, as far as possibly, unconfounded of effects of self-selections. While it is hard to fully disentangle selection and influence in observational data (see [1]), the above instrumental variable (IV) approach allows me to get closer to an estimate of this effect.

Conceive of this problem in a potential outcomes framework with boundary violation as a "treatment." Instrumenting boundary violation with the prevalence of boundary violating others at school then yields an estimate for the local average treatment effect for *compliers*; that is, it recovers the average effect of boundary violation on drinking for all adolescents who "chose" to have boundary violating friends *because* of the availability of such friends at school, net of effects for adolescents who would have self-selected into such friendships due to other, possibly "environmental" reasons which might or might not be related to the availability of boundary violating others at school and/or their drinking behavior (see [2]; related [3], and for continuous instruments, see [4], also [5]).[1]

To demonstrate how this works, I turn to simulations. Simulations allow constructing a world that resembles the above problem set up and in which we know what the right answer is that our estimation approach should recover. Moreover, by varying structural dependencies in this world, we can test the robustness of the chosen estimation approach.

---

[1] This holds under the assumption that no adolescents react opposed to their treatment assignment; that is, no adolescents exist that *refrain* from befriending boundary violating others *because* those are more prevalent at their school (so-called "monotonicity" or "non-defiers" assumption, see [6]; for proof that IVs are valid under weaker versions of this assumption, see [7]).

Consider a (simulated) world in which we observe the drinking behavior *y* of two kinds of adolescents — *compliers* and *non-compliers*.[2] Both kinds of adolescents choose whether to be friends with boundary violating others partly randomly and, with a probability *b1*, due to factors that also define their drinking behavior or factors related to those factors (this includes environmental factors such as parental background, local interpretations of religious prescriptions, etc.). However, in befriending boundary violating others, only compliers are also sensitive to the availability of such others at school (the instrument), that is, unlike non-compliers, compliers choose whether to be friends with boundary violating others also due to their prevalence at school with a probability *b3*. For a graphical representation of this situation, see Fig B1.

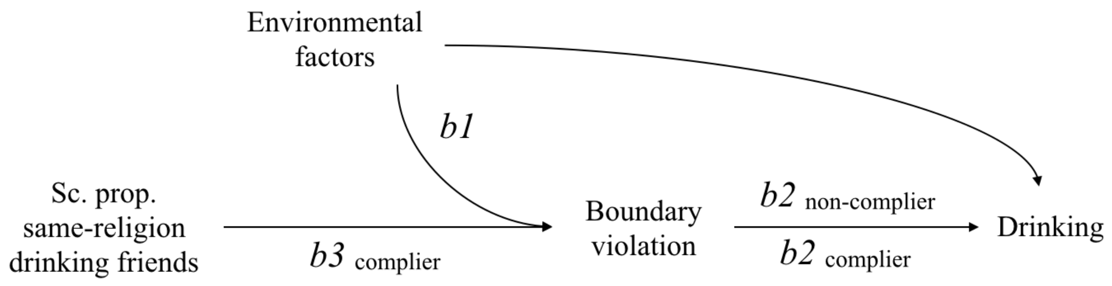

Fig B1. Direct acyclic graph for the simulated world.

The drinking behavior *y* of compliers and non-compliers is partly random, partly shaped by unobserved factors, and partly dependent on whether they have boundary violating friends (*bv*) at rates $b2_{\text{compliers}}$ and $b2_{\text{non-compliers}}$ respectively. As a result, we might therefore observe an association between having boundary violating friends and drinking in this world because some adolescents self-select into boundary violating friendships for reasons that also

[2] For the chosen IV approach to be valid, two further kinds of adolescents might exist — those who always and those who never choose to be friends with boundary violating friends, irrespectively of their prevalence of such potential friends at school ("always-takers"/"never-takers"). For simplicity, I have chosen to omit those from the set up since their existence would not alter results. (Of course, we still have to assume no "defiers".)

shape their drinking behavior and even though having such friends itself has no effect on their drinking behavior.

I simulate scenarios with $N = 5*10^6$ adolescents. I fix *b3,* compliers' sensitivity to the prevalence of boundary violating others at school in befriending boundary violating others, at 0.5.[3] Moreover, I fix $b2_{\text{compliers}}$ at 0.3, a rate comparable to the one found in the above analysis. I then implement various combinations of *b1* and $b2_{non\text{-}compliers}$, including $b2_{non\text{-}compliers}$ being zero.

Results in Table B1 demonstrate that the IV approach recovers the average effect of boundary violation on drinking among the compliers — i.e., the effect of having boundary violating friends on drinking among those who drink *because* they hold such friendships due to the prevalence of boundary violating others at school; moreover, it does so consistently, no matter how strongly other (possibly environmental) factors that might be related their drinking behavior pushes them into having boundary violating friends or how strongly drinking among non-compliers is affected by having boundary violating friends.

While the IV approach thus does exactly what is needed to answer the theoretical question posed in this paper, it is important to emphasize limitations that might weigh heavy given other, especially social policy purposes. For example, it is generally impossible to identify *who* are compliers/non-compliers and (without making strong assumptions) the estimated effect is not informative about the effect of boundary violations over the whole population (see [8], also [9]).

In sum, the simulations demonstrate that the IV approach is well-suited to provide additional plausibility for interpreting the estimated effect of boundary violation on drinking as social influence. While this is (arguably) the best we can do with observational data, we cannot ultimately be sure of this interpretation since the IV approach, of course, does not "prove" causality. In particular, I cannot rule out that some environmental factors influence drinking *and* the proportion of same-religion drinking friends at school *beyond* the proportion of same-religion friends at school and the proportion of drinking friends at school.

---

[3] Results are substantial the same for rates of 1.0, 1.5, and 2.0.

Table B1. Simulation results for estimators of boundary violations (*bv*) on drinking (*y*) given varying structural dependencies for *compliers* / *non-compliers*.

$b2_{\text{non–complier}}$	$b1$	$b2_{\text{complier}}$	y ← bv	bv ← env	$cov_{(\text{y,bv})}$	var_{y}	var_{bv}
0.0	0.0	0.3	0.29	0.09	–0.03	1.93	0.24
0.1	0.0	0.3	0.31	0.09	–0.03	1.95	0.24
0.5	0.0	0.3	0.30	0.09	0.03	2.06	0.24
1.0	0.0	0.3	0.31	0.09	0.09	2.29	0.24
2.0	0.0	0.3	0.31	0.09	0.21	3.01	0.24
0.0	0.1	0.3	0.29	0.09	0.00	1.92	0.24
0.1	0.1	0.3	0.29	0.09	0.01	1.94	0.24
0.5	0.1	0.3	0.31	0.09	0.06	2.07	0.24
1.0	0.1	0.3	0.30	0.09	0.13	2.32	0.24
2.0	0.1	0.3	0.30	0.09	0.26	3.10	0.24
0.0	0.5	0.3	0.30	0.08	0.13	1.88	0.24
0.1	0.5	0.3	0.29	0.08	0.14	1.92	0.24
0.5	0.5	0.3	0.31	0.08	0.20	2.10	0.24
1.0	0.5	0.3	0.29	0.08	0.28	2.43	0.24
2.0	0.5	0.3	0.30	0.08	0.42	3.40	0.24
0.0	1.0	0.3	0.30	0.07	0.23	1.85	0.25
0.1	1.0	0.3	0.30	0.07	0.24	1.89	0.25
0.5	1.0	0.3	0.31	0.07	0.31	2.12	0.25
1.0	1.0	0.3	0.30	0.07	0.39	2.51	0.25
2.0	1.0	0.3	0.29	0.07	0.56	3.61	0.25
0.0	2.0	0.3	0.29	0.04	0.30	1.83	0.25
0.1	2.0	0.3	0.30	0.04	0.32	1.88	0.25
0.5	2.0	0.3	0.30	0.04	0.39	2.14	0.25
1.0	2.0	0.3	0.29	0.04	0.48	2.57	0.25
2.0	2.0	0.3	0.30	0.04	0.66	3.76	0.25

$N = 5*10^6$

Appendix C: Results of logistic regression for the single equation models of drinking.

Above, I had chosen to present results from ordinary least square regressions (OLS) predicting a binary outcome (drinking), While this choice was necessary to yield reliable estimates for the dual equation system and enabled me to directly compare estimates between the single and dual equation system, it is not without shortcomings. In particular, the linear probability model can predict probabilities out of limit; moreover, it rests on the assumption that for one unit change in the regressors there is a constant change in the predicted probability. Given this, I present results of a logistic regression for the single equation models of drinking in Table C1. Results confirm the patterns found in the main analysis.

Table C1: Complete logistic regression models of drinking.

	(1) AddVector	(2) Naive	(3) Combined	(4) Controls I	(5) Controls II	(6) +JointDist
Same-religion friends	–0.18***		–0.27***	–0.32***	–0.32***	
	(3.75)		(5.11)	(8.36)	(8.44)	
Drinking friends	0.34***		0.28***	0.16***	0.16***	
	(8.65)		(9.80)	(6.13)	(6.14)	
Boundary violation (BV)		0.53***	0.59***	0.58***	0.77***	0.83***
		(8.34)	(8.75)	(8.77)	(7.81)	(6.10)
Same-religion * drinking friends				0.04***	0.04***	
				(4.33)	(4.06)	
Female				–0.09	–0.09	–0.08
				(1.55)	(1.56)	(1.67)
Age				0.17***	0.18***	0.17***
				(5.10)	(5.11)	(5.03)
Religious attendance				–0.23***	–0.22***	–0.23***
				(5.25)	(5.22)	(5.60)
R: Conservative protestant				ref.	ref.	ref.
R: Mainline protestant				–0.00	0.21*	0.19
				(0.05)	(2.02)	(1.92)
R: Catholic				0.22***	0.36***	0.37***
				(3.73)	(3.73)	(4.05)
BV * Conservative protestant					ref.	ref.
BV * Mainline protestant					–0.68**	–0.52
					(2.95)	(1.81)
BV * Catholic					–0.28*	–0.31*
					(2.00)	(2.42)
Interview wave	–0.42***	–0.45***	–0.39***	–0.46***	–0.45***	–0.45***
	(4.25)	(4.39)	(3.97)	(5.84)	(5.78)	(5.79)
Intercept	0.48***	0.55***	0.37***	–1.87***	–2.03***	–2.23***
	(4.44)	(6.46)	(3.47)	(3.48)	(3.52)	(3.76)
Joint dist. dummies	No	No	No	No	No	Yes
N	4510	4510	4510	4510	4510	4469

Note: Sample is limited to religious adolescents who specified belonging to Conservative Protestant, Mainline Protestant, or Catholic religious traditions. All models control for interview wave. Models 4 to 6 also control for sex, age, religious tradition, religious attendance, the product of the number of same-religion friends and the number of drinking friends. Models 5 and 6 control for the interaction between religious traditions and boundary violation status. Model 6 includes indicator variables for all empirical combinations of the number of same-religion friends and the number of drinking friends.

Absolute z statistics in parentheses; robust standard errors, clustered within schools.

* $p < 0.05$, ** $p < 0.01$, *** $p < 0.001$ (two-tailed tests).

References in S1 Appendices.

[1] Shalizi CR, Thomas AC. Homophily and contagion are generically confounded in observational social network studies. Sociological Methods & Research. 2011;40(2):211-39.

[2] Angrist JD, Imbens GW, Rubin DB. Identification of causal effects using instrumental variables. Journal of the American Statistical Association. 1996;91(434):444-55.

[3] Imbens G. Instrumental Variables: An Econometrician's Perspective (No. w19983). National Bureau of Economic Research. 2014.

[4] Angrist JD, Imbens GW. Two-stage least squares estimation of average causal effects in models with variable treatment intensity. Journal of the American Statistical Association. 1995;90(430):431-42.

[5] Björklund A, Moffitt R. The estimation of wage gains and welfare gains in self-selection models. The Review of Economics and Statistics. 1987:42-9.

[6] Imbens, GW, Angrist JD. Identification and estimation of local average treatment effects. Econometrica. 1994;62(2):467-75.

[7] De Chaisemartin C. Tolerating defiance? Local average treatment effects without monotonicity. Quantitative Economics. 2017;8(2):367-96.

[8] Winship C, Sobel, M Causal Inference in Sociological Studies. In: Hardy M, Bryman, A, editors. Handbook of Data Analysis. London, UK: Sage Publications. 2004:481-503.

[9] Angrist JD, Evans WN. Children and their parents' labor supply: Evidence from exogenous variation in family size. National Bureau of Economic Research. 1996; w5778.
